# Pre-operative visualization of cerebral vasculature using photon-counting CT ultra-high resolution pure lumen imaging

**DOI:** 10.1016/j.bas.2026.107451

**Published:** 2026-09-25

**Authors:** Victor Volovici, Yasmin Sadigh, Marcel Dijkshoorn, Anke van der Eerden, Renske Gahrmann

**Affiliations:** a Department of Neurosurgery, Erasmus MC Stroke Center, Erasmus University Medical Center, Rotterdam, the Netherlands; b Center for Complex Microvascular Surgery, Erasmus University Medical Center, Rotterdam, the Netherlands; c Department of Radiology and Nuclear Medicine, Erasmus Medical Center, Rotterdam, the Netherlands

**Keywords:** Preoperative imaging, Photon-counting CT, Neuronavigation, Neurosurgery, Skull base surgery, Vascular neurosurgery

## Abstract

**Introduction:**

Preoperative neuronavigation relies on multimodal imaging to delineate lesion anatomy and surrounding neurovascular structures. Ultra-high-resolution photon-counting CT (UHR PCCT) provides submillimeter vascular and osseous detail and may improve surgical planning compared with conventional CT and MRI.

**Research question:**

To evaluate the potential added value of UHR PCCT for preoperative neuronavigation in selected complex intracranial lesions.

**Materials and methods:**

We performed a selected retrospective observational pilot study of patients who underwent preoperative PCCT angiography and/or venography for neuronavigation between 2023 and 2026 at a tertiary neurosurgical center. Of 45 examinations, twenty patients were selected based on lesion complexity, vascular involvement, and size. PCCT findings were compared with conventional CT and MRI. Operative reports were retrospectively reviewed for intraoperative use of PCCT-derived findings.

**Results:**

The cohort comprised twelve meningiomas, two aneurysms, one arteriovenous malformation, and five other intracranial lesions. Median age was 53 years (IQR 46-63), and 80% were female. PCCT demonstrated additional arterial anatomical information in eleven patients (55%), including perforators in two (10%), and additional tumor vascular supply in seven patients (35%). Additional venous anatomical information was identified in six and skull base, orbital, or osseous findings in eight (40%) patients. The additional PCCTA findings were used intraoperatively in all patients.

**Discussion and conclusion:**

UHR PCCT demonstrates substantial potential as a comprehensive imaging modality for neurosurgical navigation and provides additional neurovascular and osseous anatomical information in this selected cohort. The definitive intraoperative use of these findings merits prospective comparative validation of PCCT as a complementary preoperative imaging modality.

## Highlights

• UHR PCCT depicts arterial, venous, and osseous anatomy for neuronavigation.
• PCCT improves visualization of vascular anatomy versus conventional imaging.
• Added value of UHR PCCT was greatest in skull base meningiomas and vascular lesions.
• Pure-lumen reconstructions enhance depiction of small-caliber vessels.
• PCCT shows potential for comprehensive neuronavigation imaging.

## 1 Introduction

Preoperative imaging for navigation purposes has been a staple of preoperative planning since the widespread introduction of neuronavigation in the late 1990s (Watanabe et al., 1987). Although never intended as a substitute for thorough anatomical knowledge, neuronavigation was initially developed as an adjunct to facilitate smaller, more precise craniotomies (Orringer et al., 2012; Enchev, 2009). Over time, it has become standard of care for most neurosurgical procedures (Orringer et al., 2012; Enchev, 2009).

In recent decades, neuronavigation has evolved substantially (Enchev, 2009). Modern systems integrate multimodal (MR/CT) imaging and allow for increasingly sophisticated preoperative planning. These include the visualization of white matter tracts through high throughput tractography (Pujol et al., 1996; Tharin and Golby, 2007), the visualization of adjacent neurovascular structures, and improved 3D understanding of the anatomical relationships between lesions and surrounding structures (Kikinis et al., 1996). These advances, together with the evolving imaging techniques, such as 3 T MRI, MR angiography, MR venography, provide an unprecedented level of anatomical detail, contributing to incremental improvements in surgical precision (Kapsalaki et al., 2012; Choi et al., 2007).

A recent addition to the imaging armamentarium is photon counting CT (PCCT), which introduces a fundamental shift in CT detector technology (Willemink et al., 2018). Photon-counting CT angiography (CTA) and venography (CTV) can be reconstructed at ultra-high resolution (UHR), achieving voxel sizes as small as 0.2 mm, offering higher spatial resolution of vascular and bony structures (Symons et al., 2018; Douek et al., 2023). This level of detail proves particularly valuable in complex cases, such as skull base meningiomas, highly vascularized lesions, or lesions that encase or displace critical vascular structures, including perforating arteries (Pourmorteza et al., 2017; Benson et al., 2024; Sadigh et al., 2026).

Meningiomas display variability in vascularity, with some lesions showing extensive intra-tumoral arterial networks. Tumor hypervascularity correlates with histological subtype, tumor size, and location, and has direct implications for surgical planning, as highly vascularized tumors may increase the risk of intraoperative bleeding and complicate resection (Ungureanu et al., 2025). A small subset of meningiomas demonstrates intraosseous or primary extradural growth, most frequently affecting the skull base. Although rare, representing approximately 1-2% of all meningiomas, intraosseous meningiomas pose challenges for preoperative imaging and surgical access due to their intimate association with bone and adjacent vascular structures (Agrawal et al., 2007; Tokgoz et al., 2005).

Given the availability of PCCT at the Erasmus MC University Medical Center Rotterdam, and our promising initial experience with its high-resolution and pure-lumen CTA reconstructions in pre-operative planning, we designed this study as a pilot retrospective evaluation of patients who underwent pre-operative PCCT neuronavigation imaging as part of routine clinical care. Our primary anticipated application was in complex skull base meningiomas, in which detailed delineation of osseous and neurovascular anatomy may be particularly relevant for surgical planning. We nevertheless included a heterogeneous group of intracranial lesions to explore the extent to which the potential anatomical advantages of PCCT could be transferred to other pathologies, including vascular lesions and intra-axial tumors, where conventional imaging, particularly MRI, remains central to preoperative characterization.

The objective of this study was to evaluate the potential value of PCCT in preoperative assessment by determining whether ultra-high-resolution PCCT provides additional neurovascular and osseous anatomical information compared with standard MRI and CT.

## 2 Materials and Methods

### 2.1 Eligibility criteria

Patients who underwent neurosurgical procedures requiring preoperative neuronavigation scanning with either UHR CTA and/or CTV on PCCT between 2023 and 2026 for various pathologies were reviewed. Patients who underwent cerebral revascularization surgery were excluded. Cases were selected based on the following criteria: the presence of prominent vascular structures (arterial and/or venous) within or surrounding the lesion; lesion size, with larger lesions prioritized; availability of additional imaging (MRI or CT neuronavigation studies following standard institutional protocols) to allow for comparison; and a variety of pathological diagnoses, ensuring that the study sample was not limited to meningiomas alone. A comparison was made between PCCT and conventional imaging modalities to evaluate the added value of pre-operative PCCT over conventional neuronavigation post-contrast CT and MRI. Inclusion was limited to scenarios in which high-resolution vascular imaging was expected to influence surgical planning. These were typically lesions with complex vascular anatomy and osseous or foraminal involvement. If PCCT did not provide additional information beyond existing imaging, these cases were not included. This retrospective study was deemed not subject to the Dutch Medical Research Involving Human Subjects Act (WMO) following review by the local Medical Ethics Review Committee (METC) of the Erasmus MC. This study was conducted in accordance with the Declaration of Helsinki and adheres to the Strengthening the Reporting of Observational Studies in Epidemiology (STROBE) guidelines (von et al., 2008).

### 2.2 CT angiography acquisition and reconstruction

CTA scans were performed using a dual-source photon-counting CT system (NAEOTOM Alpha, Siemens Healthineers, Forchheim, Germany). Scans were acquired in helical mode with a tube voltage of 120 kVp using automatic tube current modulation (CARE Dose4D and CARE keV; IQ level 90-145), with a rotation time of 0.25s and a pitch of 0.55-0.80. Detector collimation was 0.2 mm (total collimation 24 mm), enabling ultra-high-resolution imaging. Although the system has two X-ray tubes, the acquisition was performed with a single tube and fixed voltage, with spectral information derived from PCCT detector technology rather than conventional dual-energy CT.

Images were reconstructed with a slice thickness of 0.2-0.4 mm and isotropic voxel size down to 0.2 mm, using ultra-high-resolution kernels (Hv, Hr, Qr) with iterative reconstruction strength (Q3-Q4). Reconstruction included a low-noise and UHR series at a virtual mono-energetic level of 55 keV, and a spectral postprocessing dataset primarily displayed monoenergetic images at 40 keV, and bone-subtracted angiographic pure lumen images. For the comparative assessment, all patients were evaluated using the same reading series. All patients were reconstructed with the same core protocol.

Intravenous contrast consisted of 80 mL iodixanol (Visipaque®, 320 mg iodine/mL, GE Healthcare) administered at 5.0 mL/s, followed by a 40 mL saline flush. Bolus tracking was used for scan initiation.

Across the study cohort, the UHR CTA acquisition resulted in a mean CTDIvol of 18.2 mGy and mean DLP of 352 mGy cm. A standard neuronavigation CT acquisition at our institution is performed at 120 kVp (effective mAs 300), yielding a CTDIvol of 50.6 mGy and DLP of 1046 mGy cm.

### 2.3 Data collection

For all patients, patient characteristics and lesion characteristics were extracted from electronic medical records, including sex, age at treatment, indication for surgery, and histopathological diagnosis. Involvement of vascular structures (arterial and/or venous), lesion size (maximal axial diameter in mm), and lesion location were reviewed based on available imaging. All PCCT and comparative conventional imaging were first interpreted in routine clinical workflow by board-certified neuroradiologists or by a neuroradiology fellow, with radiology residents contributing under direct supervision. These clinical reports represent the initial assessment before surgical intervention (and thus blinded for clinical outcome). Because no standardized reporting template exists for neuronavigation imaging at our institution, all examinations included in the study were subsequently re-reviewed in a structured manner by one neuroradiologist (R.G., 5 years of experience in neurovascular imaging) and by the operating neurosurgeon (V.V.). PCCT and conventional studies were reviewed side by side. Reviewers were not blinded to clinical information or, given the retrospective design, to the surgical findings. An additional finding was recorded when a pre-specified anatomical item was identifiable on PCCT but not identifiable, or not identifiable with diagnostic confidence, on the conventional imaging available for that patient. The pre-specified items were arterial feeders and their vessel of origin, perforating arteries, arterial variants, venous anatomy including bridging veins and sinus involvement, vessel encasement or displacement, intraforaminal tumor extension, and osseous involvement including hyperostosis and intraosseous extension. Higher conspicuity alone, a structure visible on both modalities and merely sharper on PCCT, was not counted. Assessment was performed side by side, unblinded, by a neuroradiologist (R.G.) together with the operating neurosurgeon (V.V.), with disagreement resolved by consensus. The surgical relevance judgement was made by the operating surgeon alone and is reported separately. Operative reports for all included patients were retrospectively reviewed by the operating neurosurgeon (V.V.) to determine whether findings identified on PCCTA but not on conventional pre-operative imaging were documented as having been used during the surgical procedure.

### 2.4 Statistical analysis

Descriptive statistics were used to summarize patient demographics, lesion characteristics, and additional information provided by UHR PCCTA. For continuous variables, medians and interquartile ranges (IQR) were reported. Counts were presented with percentages, with the denominator stated for every subgroup. No inferential testing was performed in this selected series. Statistical analyses were performed using SPSS software (IBM SPSS Statistics for Windows, Version 29.0.1.0.).

## 3 Results

### 3.1 Patient inclusion

A total of 45 pre-operative UHR PCCT scans were performed between March 2023 and February 2026 (Fig. 1). The majority of the scans were obtained in patients harboring meningiomas (n = 29). Among the remaining 16 patients, other intracranial tumors included astrocytoma (n = 2), dermoid cyst (n = 1), craniopharyngioma (n = 1), pineocytoma (n = 1), metastasis (n = 1), and cavernoma (n = 2). In addition, six vascular lesions were scanned: arteriovenous malformation (AVM) (n = 2), intracranial aneurysms (n = 3), and a high spinal dural AV-fistula (n = 1). Finally, one patient had osteopetrosis. Nineteen patients were excluded based on no new findings on PCCT compared to conventional imaging. Six patients were excluded as PCCT was not acquired for preoperative neuronavigation. Ultimately, twenty patients were included in this study. To reduce overrepresentation of a single disease entity, the final selection of 20 cases was chosen to broadly reflect this distribution. The final cohort comprised 12 meningiomas (60%) and 8 non-meningioma cases (40%). Selection was further guided by the anticipated relevance of high-resolution vascular and osseous imaging for neurosurgical planning. Prior to PCCT, nineteen patients underwent MRI, most commonly obtained outside the tertiary referral center (n = 13). Almost all MRI protocols included 3D T1-weighted post-contrast imaging (n = 18), T2-weighted imaging (n = 17), T2-FLAIR imaging (n = 15), and DWI (n = 13). Less commonly acquired were SWI or T2*-GRE (n = 13), DSC perfusion (n = 1), MRA (n = 2), CISS imaging (n = 1), and fMRI (n = 1) sequences. The DSC-perfusion and fMRI were performed in case #18 with an astrocytoma. One patient underwent DSA without prior available MRI.

**Fig. 1:**
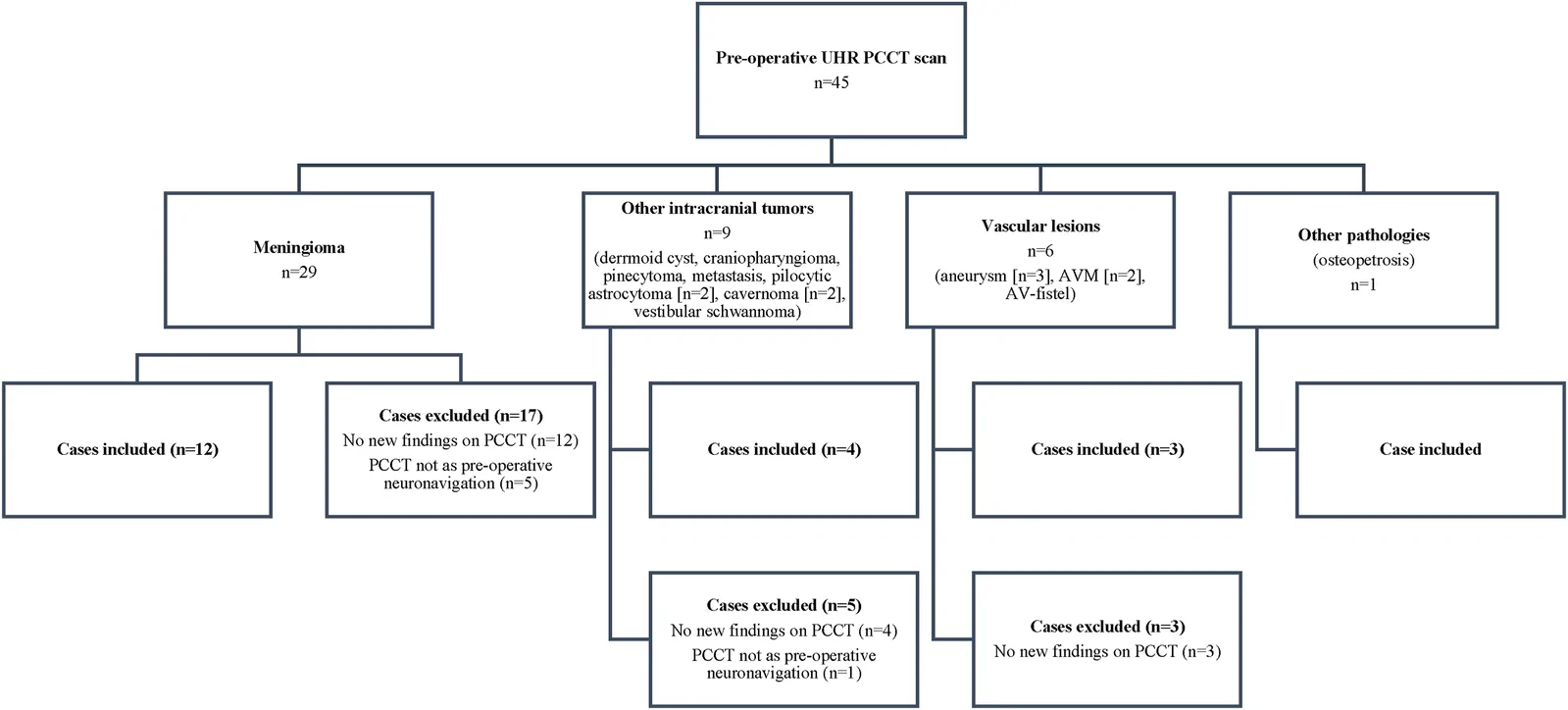
Flowchart of patient inclusion between March 2023 and February 2026. Twelve patients with meningiomas, two patients harboring aneurysms, one patient diagnosed with an AVM SM-grade III, one patient harboring an astrocytoma (WHO grade II), one patient with a dermoid cyst, one patient with a recurrent pineocytoma, and one patient with cerebral metastasis (n = 20) were included.

### 3.2 Patient and lesion characteristics

Of all patients, 80% (n = 16) were female. Median age at treatment was 53 (IQR 46-63) years. All patient and lesion characteristics can be found in Table 1. The majority of the included lesions were meningiomas (n = 12, 60%). Two (10%) patients harboring aneurysms, one (5%) patient diagnosed with an AVM SM-grade III, one (5%) patient harboring an astrocytoma (WHO grade II), one (5%) patient with a dermoid cyst, one (5%) patient with a recurrent pineocytoma, and one (5%) patient with cerebral metastasis were included. The majority of the meningiomas (n = 8/12, 67%) were located at the anterior skull base, three (25%) were located centrally. The median size of pathology was 43 mm (IQR 35-56) for tumors. ICA encasement by the lesion was present in nine (45%) of the cases. Lesions were closely related to the ophthalmic artery in two patients (10%), and other arterial branches included A1 (n = 2, 10%), Pcom (n = 1, 5%), P2 (n = 1, 5%), M1 (n = 4, 20%), M2 (n = 1, 5%), M4 branches (n = 1, 5%), ACA (n = 1, 5%), MCA (n = 1, 5%), and the lateral lenticulostriate arteries (LLSA) (n = 3, 15%). Venous involvement was observed in three patients, including the sagittal sinus (n = 1, 5%), Sylvian vein (n = 1, 5%), and Vein of Galen (n = 1, 5%). Indication for surgery was mostly vision loss or visual field deficits, present in eleven patients (55%). Other surgical indications were epilepsy, occurring in seven patients (35%), cognitive dysfunction (n = 3 15%), and mild aphasia (n = 1, 5%).

**Table 1:** Patient characteristics.

Case#	Sex	Age	Clinical Presentation	Diagnosis	Lesion size (diameter, volume)	Lesion location	Vascular structure involvement	Surgical approach
1	F	60	Progressive vision loss, cognitive dysfunction	Meningioma WHO grade II	64 mm, 88 mL	Sphenoid, lateral orbital wall	ICA, OphtA	Pterional
2	F	59	Vision loss, headache	Meningioma WHO grade I	43 mm, 28 mL	Sphenoid, lateral orbital wall	ICA	Pterional
3	M	66	Progressive vision loss	Dermoid cyst	36 mm, 10 mL	Sphenoid, lateral wall cavernous sinus	ICA encasement, lesion in contact with Pcom, P2, A1, M1	Pretemporal
4	F	57	Epilepsy, cognitive dysfunction, trigeminal neuralgia	Meningioma WHO grade I	67 mm, 91 mL	Planum sphenoidale, tuberculum sellae	ICA, A1 and RAH encasement, lesion in contact with M1	Pterional
5	M	30	Progressive vision loss, epilepsy, diplopia	Meningioma WHO grade II	38 mm, 21 mL	Spheno-orbital, lateral wall cavernous sinus	ICA encasement, lesion in contact with M1	Pretemporal
6	F	44	Progressive vision loss	Aneurysm	Giant (21 × 19 × 17 mm)	ICA paraclinoid superior wall (ophthalmic artery)	-	Pretemporal
7	F	75	Epilepsy, cognitive dysfunction	Meningioma WHO grade I	Lesion 1: 35 mm, 32 mL Lesion 2: 51 mm	Lesion 1: convexity Lesion 2: Parasagittal	Superior sagittal sinus	-
8	F	63	Epilepsy	Meningioma WHO grade I	61 mm, 99 mL	Spheno-orbital	-	Pterional
9	M	46	Vertigo, trigeminal neuralgia	Osteopetrosis, Type I, LRP5 mutation	-	-	-	Pretemporal, kawase
10	F	52	Hydrocephalus, vision loss, mass effect brain stem	Recurrent pineocytoma	37 mm, 16 mL	Pineal region	Vein of Galen	Intrahemispheral supra/transtentorial
11	F	60	Tumor growth, hemianopia	Metastasis of invasive mammary carcinoma	50 mm, 42 mL	Parieto-occipital region	-	-
12	F	53	Vision loss, exophthalmus	Meningioma WHO grade I	27 mm, 14 mL	Sphenoid, cavernous sinus	ICA encasement	Pterional, transcavernous
13	F	18	Epilepsy	AVM S-M grade III	Compact nidus (>4 cm)	Fronto-parietal (precentral gyrus)	MCA, ACA, and LLSA feeders	-
14	F	41	Epilepsy, mild aphasia	Meningioma WHO grade I	37 mm, 27 mL	Convexity, left (Sylvian fissure/Broca area)	Encasement of two M4 branches, fronto-opercular branch, Sylvian vein	Pterional
15	F	46	Vision loss	Meningioma WHO grade I	15 mm, 2 mL	Optic canal, cavernous sinus	ICA encasement, cavernous sinus superior wall infiltration	Pterional
16	F	53	Vision loss	Meningioma WHO grade I	52 mm, 61 mL	Sphenoid wing	Lesion in contact with ICA, M1	Pterional
17	F	54	Hypogonadism	Meningioma WHO grade I	45 mm, 19 mL	Sella turcica, cavernous sinus	ICA encasement	Pterional
18	M	32	Epilepsy	Astrocytoma WHO grade II	67 mm, 87 mL	Insula	Lesion in contact with LLSA	Pterional
19	F	72	Aneurysm growth	Aneurysm	Small (9 × 5 × 5 mm)	M1	LLSA, M2 branches from aneurysm sac	Pterional
20	F	58	Vision loss, exophthalmus	Meningioma WHO grade I	13 mm	Sphenoid wing	Lesion in contact with ophthalmic artery	Pterional

ACA: anterior cerebral artery, AVM: arteriovenous malformation, F: female, ICA: internal carotid artery, LLSA: lateral lenticulostriate arteries, LRP5: low-density lipoprotein receptor-related protein 5, M: male, M1: M1 segment of the middle cerebral artery, M2: M2 segment of the middle cerebral artery, M4: M4 segment of the middle cerebral artery, MCA: middle cerebral artery, OphtA: ophthalmic artery, P2: P2 segment of the posterior cerebral artery, Pcom: posterior communicating artery, S-M: Spetzler-Martin,WHO: World Health Organization.

For all included cases, multimodal imaging datasets were reviewed, including PCCTA, MRI, and conventional CT, with additional CT perfusion and digital subtraction angiography (DSA) data available in selected patients. Comprehensive lesion-specific reconstructions across all modalities are presented in the Supplementary Appendix Figs. 1–15.

### 3.3 UHR PCCTA versus conventional imaging

Compared with conventional imaging, UHR PCCTA provided additional anatomical information across all 20 cases, with the most frequent findings relating to arterial anatomy, tumor vascularity, and skull base or osseous involvement (Table 2). Additional information regarding the relationship between the lesion and adjacent arterial structures was identified in eleven cases (55%), including arterial displacement, encasement, luminal narrowing, and delineation of smaller arterial branches and perforators (n = 2). Additional characterization of tumor vascular supply was provided in seven cases (35%), including identification of probable or dominant arterial feeders, intratumoral vascularity, and vascular pedicles. The middle meningeal artery or inferolateral trunk was identified as a likely or dominant arterial feeder in six cases (30%), all involving meningiomas. Additional venous anatomical information was identified in six cases (30%), including characterization of cortical and emissary venous drainage and relationships with the basal vein of Rosenthal, vein of Galen, and rectus sinus.

**Table 2:** Additional anatomical findings identified by PCCT compared with conventional imaging.

Case #	Diagnosis	Conventional comparator	Findings on conventional imaging	Additional findings on PCCT	Potential surgically relevant findings
1	Meningioma WHO grade II	MRI	Right frontal extra-axial mass (6.3 cm) with contrast-enhancement, broad dural tail, and CSF cleft sign. Mild edema and marked mass effect.	Orbital roof, lateral orbital wall, and partial clinoid hyperostosis, resulting in optic nerve compression. Prominent vascular structures anterior and posterior within the mass. Displacement of the carotid terminus, ACA, and MCA branches without vascular encasement.	Vascular displacement without encasement and optic nerve compression may facilitate surgical planning.
2	Meningioma WHO grade I	MRI	Right frontotemporal extra-axial mass (4.0 cm) with broad dural tail and sphenoid bone remodeling. Mild temporal edema and mass effect. Close relation to the superior orbital fissure, foramen rotundum, foramen ovale, and optic pathway. Superior displacement of the MCA.	Likely partial supply from the right MMA, which courses lateral to the tumor. Marked superior displacement of the MCA. Mild greater sphenoid wing scalloping.	Detailed delineation of skull base foraminal involvement and adjacent neurovascular anatomy.
3	Dermoid cyst	MRI	Right temporal skull base extra-axial lesion (4.0 cm) with spontaneous T1 hyperintensity and no contrast enhancement. Mild mass effect on the temporal lobe, adjacent central vessels, and prechiasmatic optic nerve. No edema.	Broad contact with the right ICA terminus, A1, Pcom, P2, and M1/M2 branches. Close relationship to the cavernous sinus, superior orbital fissure, and right optic tract. Coarse mural calcifications. No evidence of rupture.	Detailed delineation of the relationship of the lesion to Cirkel of Willis anatomy.
4	Meningioma WHO grade I	MRI	Left frontal extra-axial mass (6.5 cm), showing enhancement and a CSF cleft. Mild mass effect and somewhat disproportionate peritumoral edema. Close relationship to the left carotid siphon, ACA, and MCA.	Encasement of the distal left ICA and encasement with focal narrowing of the left A1. The left and right A2 are displaced to the right, while the left M1 is displaced inferiorly. Recruited intratumoral/peritumoral vessels are present, with the ILT identified as a feeding artery.	Anterior circulation involvement increases complexity. Risk of perforator injury. ILT likely feeder.
5	Meningioma WHO grade II	MRI	Right sphenoid wing/clinoidal extra-axial mass (3.8 cm) with extensive skull base involvement and peritumoral edema. Extension into the cavernous sinus, anterior Meckel's cave, superior orbital fissure, foramen rotundum and likely the optic canal. Close relation to the optic nerve, ophthalmic artery, and ICA.	Close relationship to the ophthalmic artery, encasement of extradural ICA in the cavernous sinus and intradural ICA up to the carotid terminus. Superior displacement of the M1. Hypoplastic right Pcom close to the lesion. Likely arterial supply from the ILT. Prominent superolateral peritumoral venous plexus.	Extensive skull base extension with optic nerve, ophthalmic artery, carotid terminus, and M1 involvement. ILT likely feeder.
6	Aneurysm	DSA	Patent Acom complex. Right supraclinoid/supra-ophthalmic ICA aneurysm (2.0 cm) with posterior neck bleb arising from the superior wall. Relatively narrow neck (0.7 cm). No other aneurysms identified	Double Acom complex. No other new findings.	Narrow neck and posterior bleb may affect clipping strategy.
7	Meningioma WHO grade I	MRI	Bifrontal anterior lesion (4.9 cm) with intraosseous, superior sagittal sinus involvement, and extension along the falx. Mild interval growth and increase in peritumoral edema and mass effect. Second right parieto-frontal lesion (3.2 cm), stable in size.	Extensive superior sagittal sinus invasion over at least 5.9 cm. Transcalvarial involvement with hyperostosis. Pronounced osseous vascularization.	SSS invasion, transcalvarial extension, and hyperostotic bone.
8	Meningioma WHO grade I	MRI	Left middle cranial fossa extra-axial mass (6.7 cm), with marked mass effect. Extensive tumor vascularity with enlarged cortical draining veins. Displacement of MCA branches.	Extensive intratumoral vascularity with hypertrophic MMA as the dominant feeder. Extensive intratumoral radial vascularity. Venous drainage predominantly by cortical veins and hypertrophic emissary veins to the pterygoid plexus. Deep venous drainage to the basal vein of Rosenthal.	Hypertrophic MMA supply and intratumoral vascularity may aid surgical planning.
9	Osteopetrosis type I, LRP5 mutation	MRI	Diffuse osteopetrosis with calvarial/skull base thickening, including bilateral petrous apex hyperostosis causing superior displacement of the trigeminal nerves. Bilateral internal auditory canal narrowing with reduced CSF around the vestibulocochlear and facial nerves. Signs of intracranial hypertension.	Diffuse osteopetrosis with additional detailed visualization of vascular structures near the trigeminal nerve within the densely sclerotic skull base.	Combined CT/MRI depiction of neurovascular and osseous anatomy aids surgical planning.
10	Recurrent pineocytoma	MRI	Pineal region mass (enhancing component 3.9 cm), consisting of solid and cystic components with enhancement of the solid portion. Marked mass effect with narrowing of the 4th ventricle with hydrocephalus. Additional infratentorial extra-axial lesion (0.7 cm).	Right PCA branch courses along the tumor margin. Small probable arterial vessel along the anterior tumor aspect, without additional definite intratumoral arterial supply. Partial encasement and leftward deviation of the internal cerebral veins near the vein of Galen. Prominent right basal vein of Rosenthal.	Detailed depiction of the internal cerebral veins and PCA branches.
11	Metastasis of invasive mammary carcinoma	Non-contrast CT, MRI	Left occipital intra-axial mass (4.8 cm), enhancing with hemorrhagic, microcystic, and solid components. Extensive edema and mass effect. Two additional enhancing lesions in the right frontal lobe (1.0 cm and 0.6 cm), also suspicious for metastasis.	Dominant arterial feeder from the left PCA entering the ventral aspect of the lesion, with additional PCA branches and nearby distal MCA branches along the tumor margins. Prominent anterior peritumoral venous drainage into the straight sinus.	Ventral PCA feeder and close relationship to the straight sinus.
12	Meningioma WHO grade I	MRI	Right medial sphenoid wing meningioma (4.0 cm) with cavernous sinus invasion and encasement and narrowing of the ICA. Close relationship to the optic nerve. Mild adjacent edema.	Right cavernous sinus meningioma with extension into the temporal compartment, and through the superior orbital fissure toward the orbital apex. Extension into the sphenoid sinus. Preservation of the sella turcica.	ICA narrowing and skull-base extension.
13	AVM S-M grade III	MRI, DSA	Left fronto-parietal AVM (4.4 cm) centered in the precentral gyrus with a compact nidus. Associated precentral gyrus volume loss without gliosis, edema, or hemorrhage. Supplies by the left MCA and ACA, including a lenticulostriate M1 feeder. Enlarged distal left ICA. Superficial venous drainage via a dominant vein to the SSS. No deep venous drainage.	Double Acom complex. No other new findings	Superficial venous drainage pattern and absence of deep drainage may aid surgical risk assessment. Deep lenticulostriate supply and precentral gyrus involvement are relevant.
14	Meningioma WHO grade I	MRI	Left frontotemporal extra-axial mass (4.2 cm) with enhancement and increased perfusion. Extensive edema, mass effect, dural tail, and temporal bone involvement. Abnormal diploic marrow signal indicates possible intraosseous extension.	MCA branches are displaced medially and posteriorly by the tumor, with a small arterial branch traversing the anterosuperior tumor margin. The tumor demonstrates a prominent central vascular pedicle and an enlarged adjacent diploic vein. Hypertrophied left MMA appears to represent the dominant arterial feeder.	Hypertrophic MMA supply, central vascular pedicle, enlarged diploic vein, and MCA displacement.
15	Meningioma WHO grade I	MRI	Left cavernous sinus meningioma (2.0 cm). Extension into the foramen rotundum, inferior and superior orbital fissures, and cranially along the anterior clinoid. Associated dural tail and sphenoid wing hyperostosis. Probable compression of the intracanalicular left optic nerve without optic nerve edema.	Subtle focal irregularity of the lateral wall of the distal left ICA at the site of tumor contact, raising concern for focal adherence. The left ophthalmic artery is patent. Associated hyperostotic and sclerotic osseous changes involve the left sphenoid wing and lateral orbital wall.	Distal ICA adherence and foraminal extension may complicate skull base dissection.
16	Meningioma WHO grade I	Non-contrast CT, MRI	Right temporal skull base extra-axial mass (5.4 cm) with marked enhancement, extensive edema, and mass effect. Extension into the choroidal fissure. Additional presumed meningiomas at the left tuberculum sellae and left frontal convexity.	Associated hyperostosis involving the sphenoid bone. Vascular pedicle arising from the ILT supplying the tumor. Clear upward displacement of the MCA. Prominent venous drainage through the sphenoid bone and via the basal vein of Rosenthal toward the vein of Galen.	ILT feeder, MCA displacement, and venous drainage pattern may affect surgical planning. .
17	Meningioma WHO grade I	MRI	Right cavernous sinus meningioma (4.5 cm) with sphenoid/anterior clinoid hyperostosis, intrasellar-suprasellar extension and optic chiasm compression. Encasement and narrowing of the right ICA. Extension into the middle cranial fossa, retroclival region, Meckel's cave, and likely foramen rotundum with possible involvement of the optical canal and superior orbital fissure. Mass effect on the right cerebral peduncle.	Encasement of the right ICA with luminal narrowing. Partial encasement of the right M1 and close relationship with the right A1. Encasement of the right Pcom with a diminutive caliber and close relationship to the proximal right PCA. Small intratumoral vascular structure, possible representing a meningeal branch or perforating vessel.	Extensive cavernous sinus/skull base involvement with ICA, Pcom, and M1 encasement. Optic pathway and Meckel's cave involvement may complicate resection.
18	Astrocytoma WHO grade II	MRI	Large left frontotemporal (insular) infiltrative glioma with T2-FLAIR mismatch sign and no contrast enhancement or diffusion restriction. Mild to moderate mass effect.	Left MCA perforators are displaced medially by the insular tumor and course along its medial margin. No prominent intratumoral arterial vascularity. Variant circle of Willis anatomy with a fetal-origin right PCA and hypoplastic/absent left PCOM.	Detailed depiction of displaced MCA perforators may aid surgical planning.
19	Aneurysm	MRI/MRA	Left MCA bifurcation aneurysm, stable in size. Newly developed lateral bleb arising from the aneurysm. No additional intracranial aneurysms were identified.	Lobulated left M1 aneurysm (0.9 cm) with an M2 branch arising from its medial aspect and looping over the aneurysm surface. Lenticulostriate perforators originate from this branch. The inferior temporal division arises immediately distal to the aneurysm. Hypoplastic left Pcom and right Pcom infundibulum.	M2 incorporation with associated perforators may complicate clip reconstruction. .
20	Meningioma WHO grade I	Non-contrast CT, MRI	Right spheno-orbital en plaque meningioma with associated extensive hyperostosis involving the temporal, sphenoid, and frontal bones. Enhancing nodular dural thickening along the anterior temporal skull base. Intraorbital extraconal component with proptosis and possible traction on the optic nerve.	Extensive transosseous involvement with (also) involvement of the zygomatic bone, parietal bone, anterior clinoid, and all walls of the right orbit. Close relation to the ophthalmic artery.	Detailed delineation of extensive orbital and skull-base hyperostosis.

Findings on both conventional and PCCT imaging stem from clinical radiology reports. ACA: anterior cerebral artery; Acom: anterior communicating artery; AVM: arteriovenous malformation; CSF: cerebrospinal fluid; CT: computed tomography; DSA: digital subtraction angiography; ICA: internal carotid artery; ILT: inferolateral trunk; LRP5: low-density lipoprotein receptor-related protein 5; M1: M1 segment of the middle cerebral artery; M2: M2 segment of the middle cerebral artery; MCA: middle cerebral artery; MMA: middle meningeal artery; MRI: magnetic resonance imaging; P2: P2 segment of the posterior cerebral artery; PCA: posterior cerebral artery; PCCT: photon-counting computed tomography; Pcom: posterior communicating artery; S-M: Spetzler-Martin; SSS: superior sagittal sinus; T1: T1-weighted; T2-FLAIR: T2-weighted fluid-attenuated inversion recovery; WHO: World Health Organization.

Additional skull base, orbital, and osseous information was identified in eight cases (40%), including hyperostosis, foraminal and orbital involvement, and transcalvarial or transosseous extension. In the patient with osteopetrosis (case 9), UHR PCCTA additionally demonstrated vascular structures adjacent to the trigeminal nerve within the densely sclerotic skull base. Applications included identification of vascular pedicles arising from the lateral orbital wall, foramen rotundum, and other skull base foramina to facilitate early devascularization, identification and tracking of fine peripheral M4 branches in sphenoid wing meningiomas, delineation of the interface between vessels and the meningioma capsule, and assessment of the distance between the free edge of falcine meningiomas and adjacent sinuses, draining veins, or venous lakes. These findings were used to inform intraoperative anatomical identification and surgical decision-making.

In the two aneurysm cases (cases 6 and 19), UHR PCCTA provided additional information regarding vascular anatomy, including a double Acom complex in one case and M2 branch incorporation with associated lenticulostriate perforators in the other. In Case 19, bone-subtracted PCCTA demonstrated an M2 branch arising directly from the aneurysm sac, with lenticulostriate perforators originating from that branch (Supplementary Fig. 14); this configuration governs clip selection and the margin for sac manipulation, and it was not resolved on TOF MRA. In Cases 5 and 17, PCCTA demonstrated tumor supply from the inferolateral trunk, and in Case 17 additionally encasement of the Pcom not visible on MRI (Supplementary Figs. 4 and 12); feeder origin from the cavernous ICA bears directly on the order of devascularization and on how far the cavernous sinus wall can be pursued.

### 3.4 Illustrative cases

Across the illustrative cases (Case 1, 8, 13, 14, and 16), multimodal imaging demonstrated complementary but non-equivalent contributions of MRI and PCCT-based CTA for preoperative neuronavigation (Fig. 2, Fig. 3, Fig. 4, Fig. 5, Fig. 6). MRI consistently provided robust delineation of lesion extent and soft-tissue characteristics, while PCCTA added anatomical detail of the lesion-vessel-bone interface that was not fully appreciable on MRI alone. In meningioma cases (Fig. 2, Fig. 3, Fig. 5, Fig. 6), UHR PCCTA and bone-subtracted reconstructions improved visualization of intratumoral and dural arterial supply, including hypertrophic ophthalmic and middle meningeal artery branches, inferolateral trunk supply, and central vascular pedicles, as well as more precise assessment of relationships with adjacent cortical vessels, venous structures, and hyperostotic bone. In the AVM case (Fig. 4), PCCT provided high-fidelity depiction of nidus architecture and its spatial relationship to feeding ACA/MCA branches and draining veins, showing good correlation with DSA while offering improved 3D anatomical context compared with standard MRI reconstructions. Across all cases, bone-subtracted and 0.2 mm isotropic PCCT reconstructions consistently enhanced visualization of surgically relevant vascular anatomy, particularly small-caliber feeders and vessel displacement patterns.

**Fig. 2:**
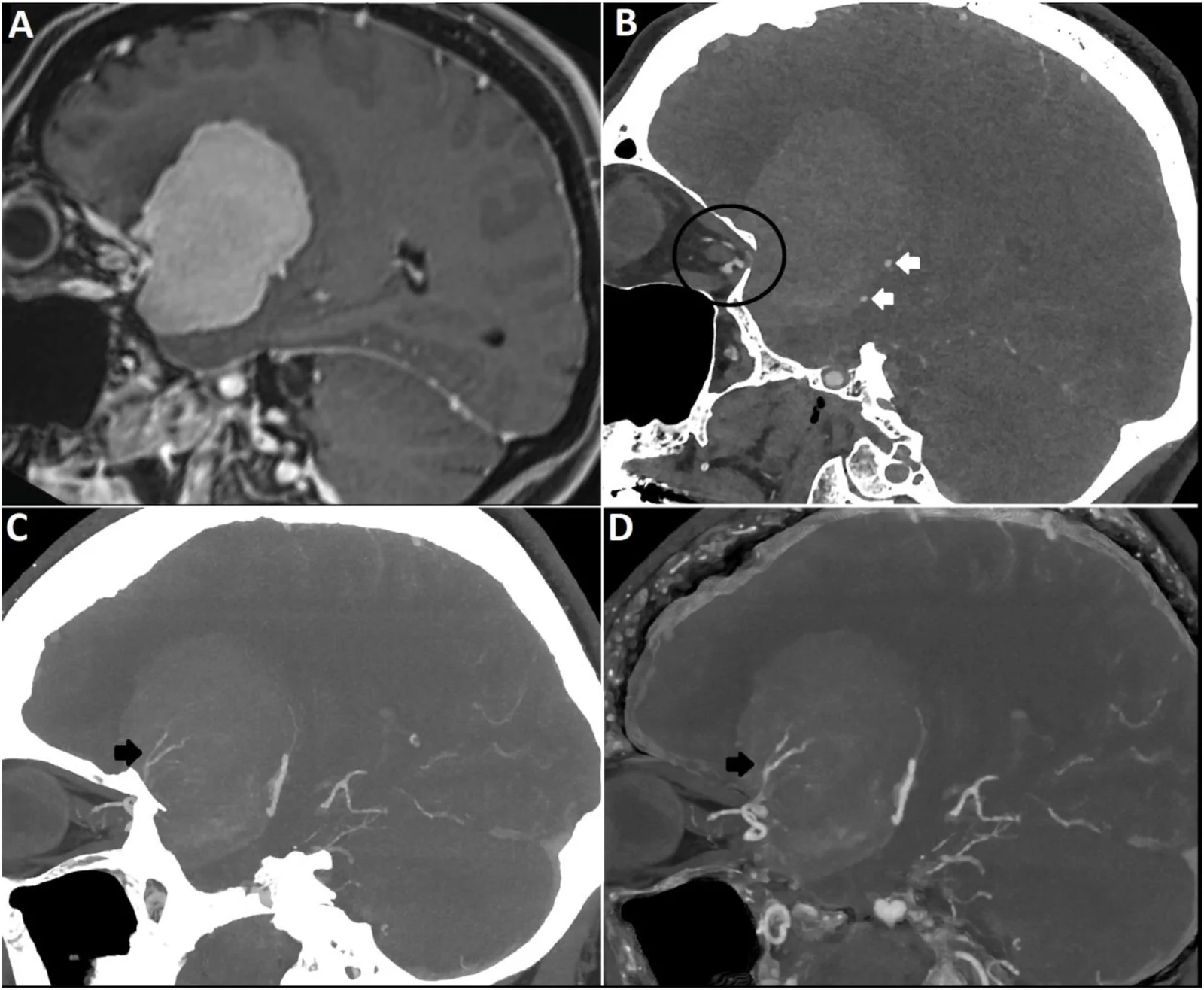
**Case #1. Sphenoid, lateral orbital wall meningioma**Post-contrast sagittal T1-weighted MRI (**A**) demonstrates a large, avidly enhancing mass, in close relation to the orbital apex. Arterial phase PCCT with 0.2 mm slice thickness (**B**) depicts the meningioma corresponding with MRI, with a hypertrophic ophthalmic artery (**circle**) and close spatial relationship to early branching M2 segments (**arrows**). 3D MIP reconstructions without (**C**) and with (**D**) bone subtraction demonstrate a prominent central vascular pedicle within the lesion, consistent with a central feeding vessel (black arrow). The anatomy of the vessel is better appreciated with bone subtraction.

**Fig. 3:**
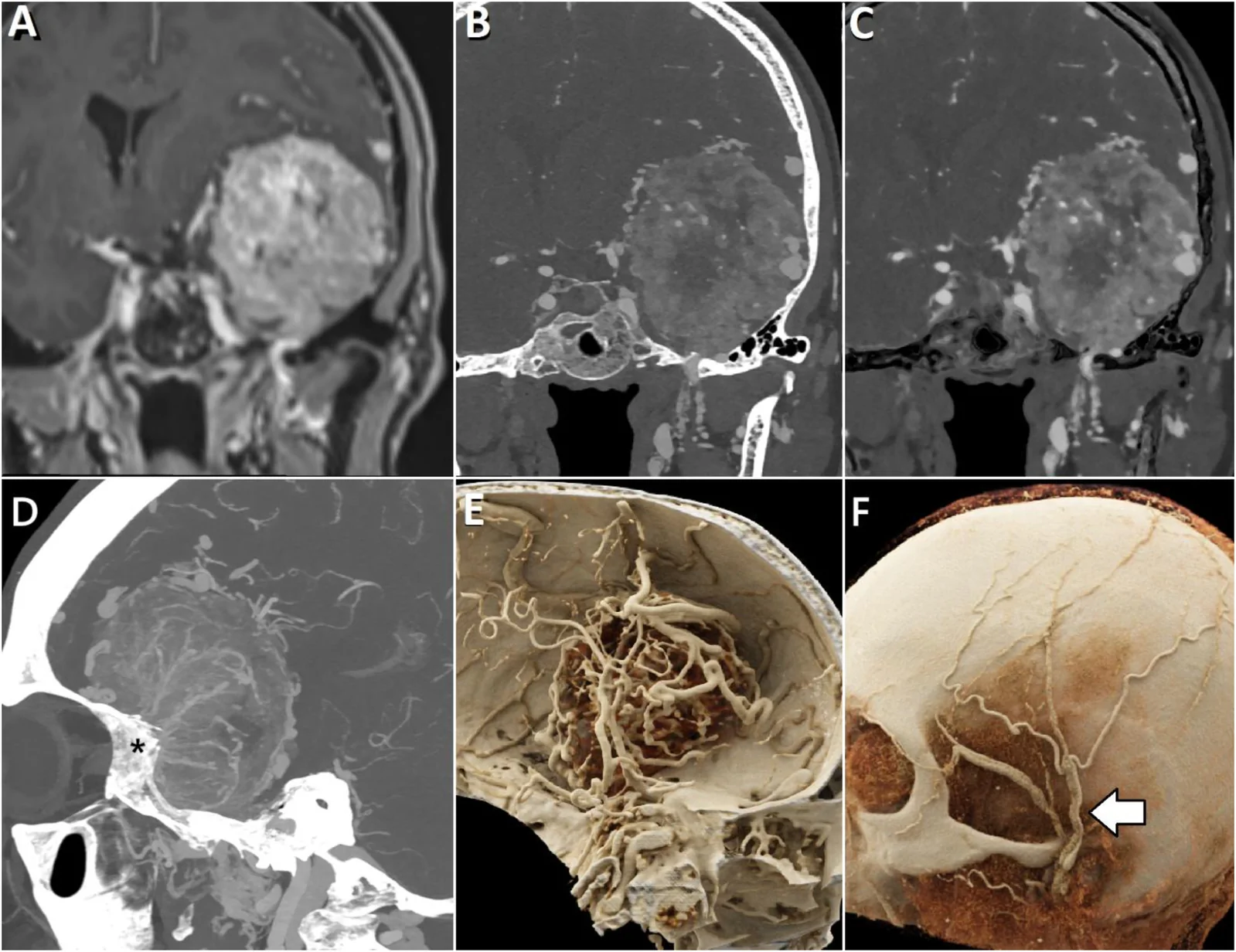
**Case #8.** Spheno-orbital meningioma. Coronal T1w post-contrast MRI (**A**), post-contrast CT (**B**), and pure-lumen reconstruction (**C**) delineate the lesion, demonstrating clearer differentiation between tumor parenchyma and vascular structures. Sagittal MIP CTA (**D**) shows extensive intratumoral radial vascularity and underlying hyperostosis (*). 3D reconstructions show tumor vascularization (**E**) and a hypertrophic middle meningeal artery (**F**: arrow).

**Fig. 4:**
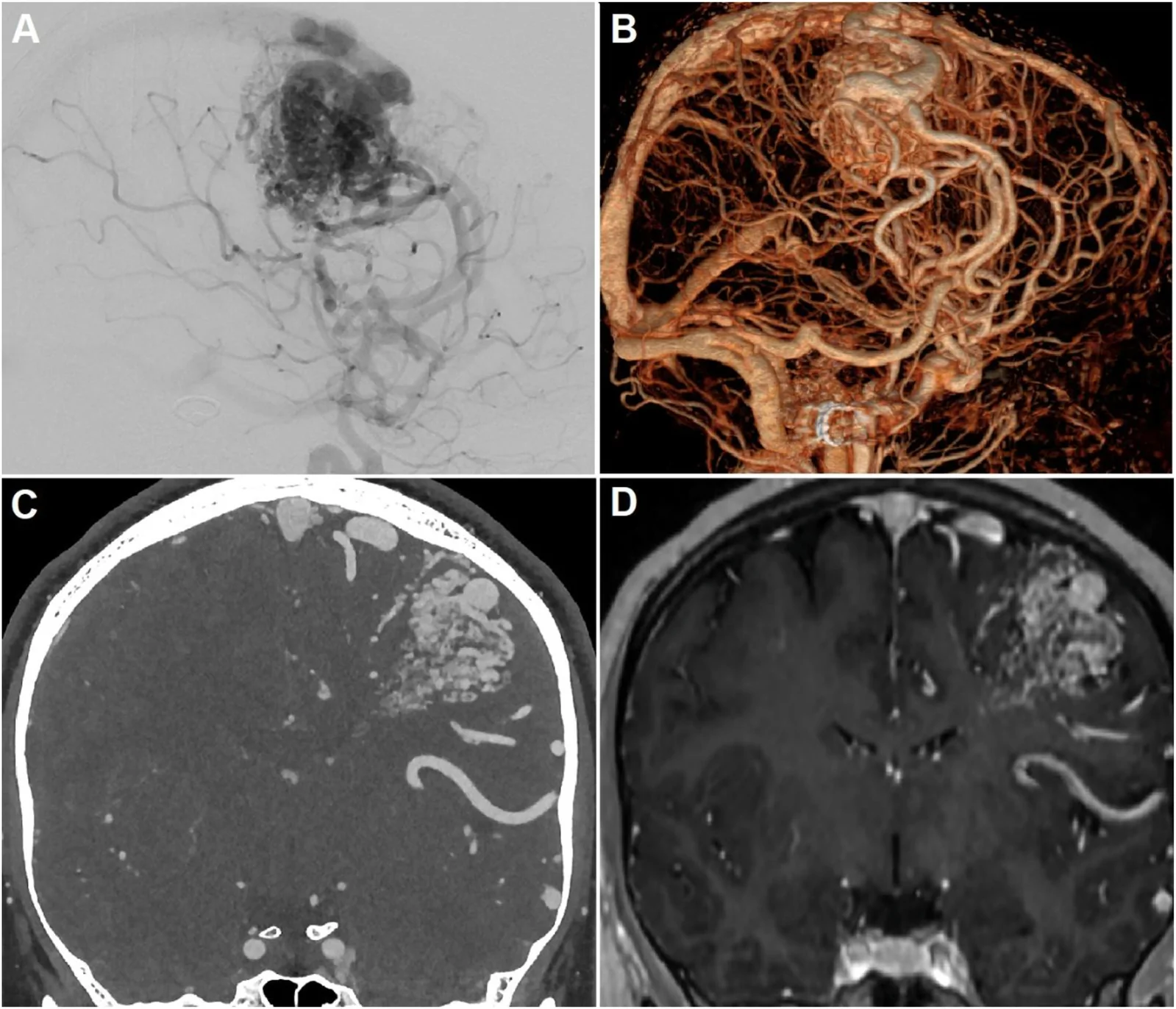
**Case #13.** Left fronto-parietal (precentral gyrus) AVM with a large (>4 cm) nidus. DSA (**A**) demonstrates the AVM nidus with arterial supply from the left ACA and MCA, and superficial venous drainage without deep venous outflow. A 3D reconstruction of contrast-enhanced PCCT (**B**) depicts the AVM in relation to the surrounding vasculature. Coronal 0.2 mm PCCT reconstruction (**C**) provides high-resolution and highly detailed delineation of the nidus and adjacent vessels, while a matched 1.6 mm post-contrast MRI reconstruction (**D**) through the same plane provides cross-modality correlation.

**Fig. 5:**
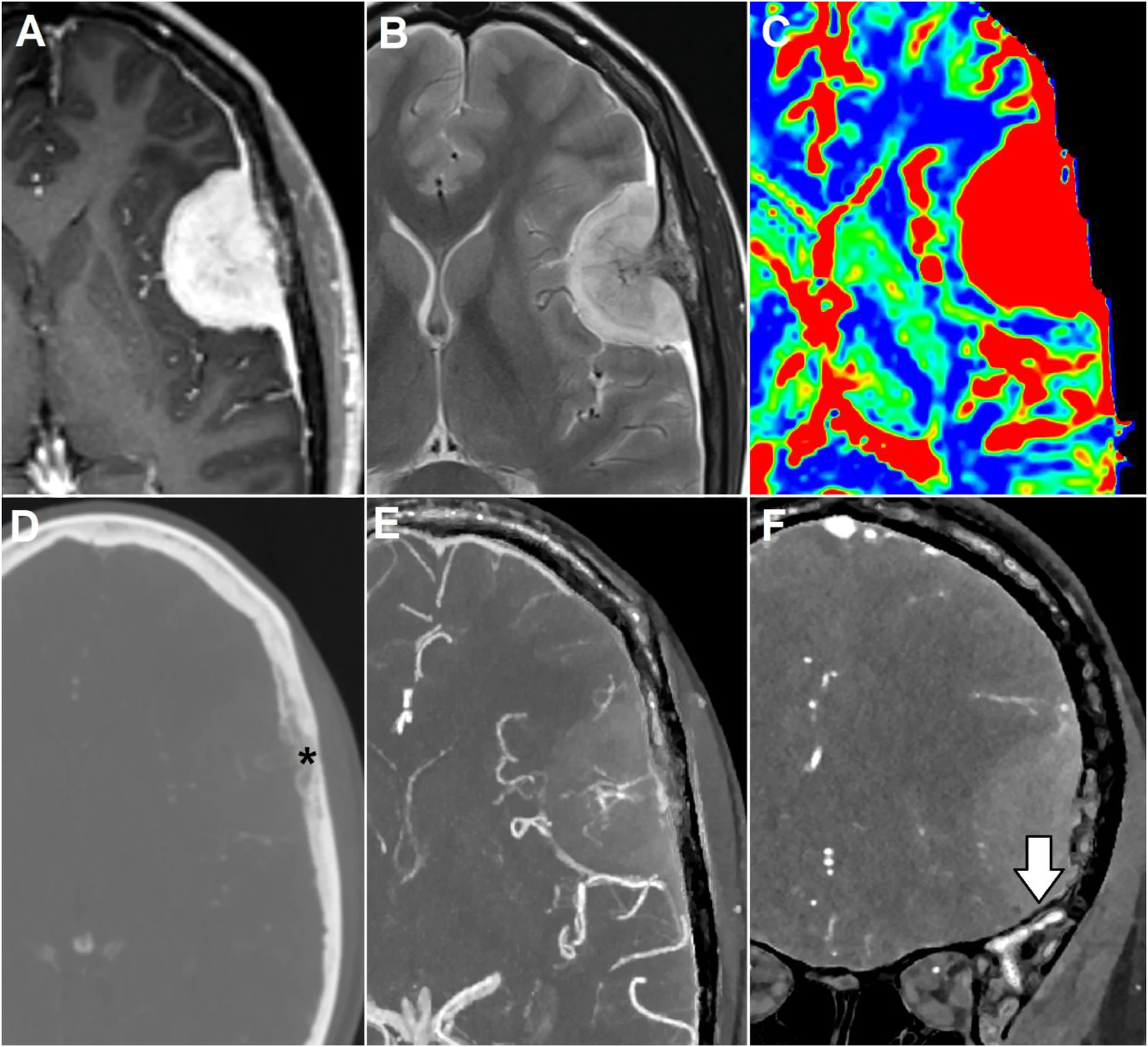
**Case #14.** Left hemispheric convexity (Sylvian fissure/Broca area) meningioma. T1w post-contrast (**A**) and T2w (**B**) MRI shows a large convexity meningioma with a dural tail. DSC-perfusion MRI demonstrates increased rCBV (**C**). Limited osseous invasion is visible on MRI and CT (**D**: *). CTA MIP with bone-subtraction (**E–F**) depict internal vascular supply (not visible on MRI), including a prominent central vascular pedicle, together with an enlarged adjacent diploic vein (**arrow**).

**Fig. 6:**
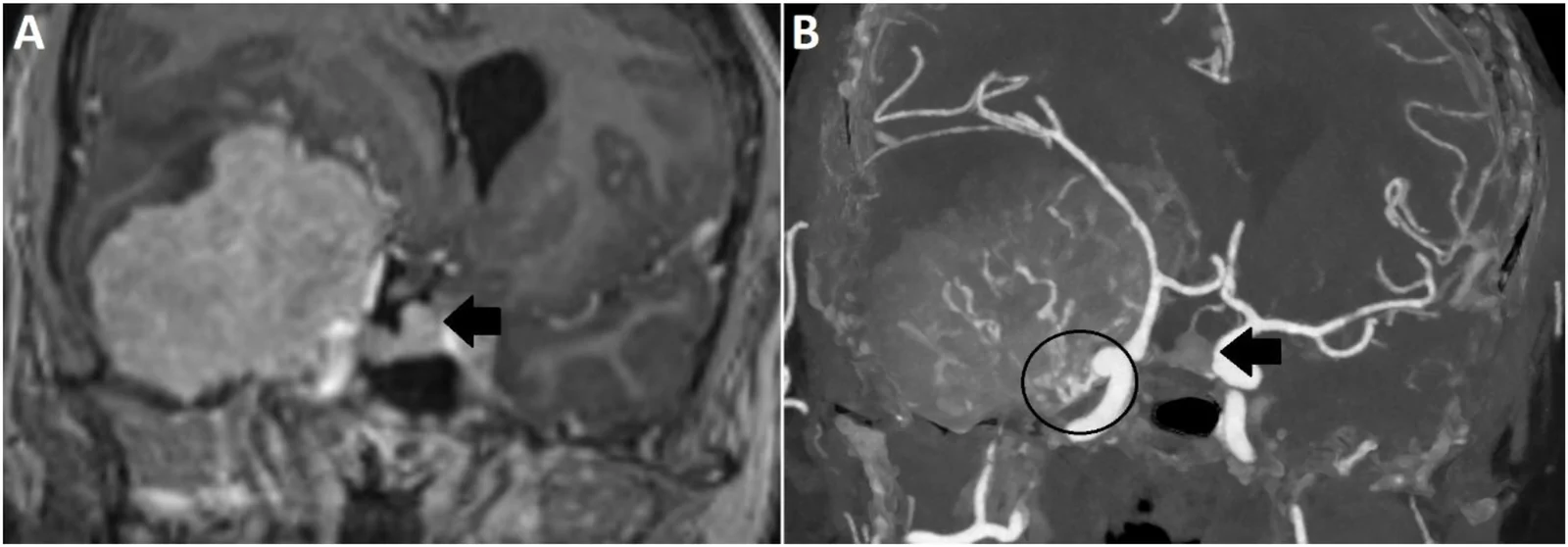
**Case #16.** Right Sphenoid wing meningioma. Coronal T1-weighted post-contrast MRI (**A**) shows a large right temporal meningioma. The bone-subtracted CTA acquired on PCCT (**B**) demonstrates the interval vascular supply via a pedicle arising from the inferolateral trunk (ILT; **circle**) and delineates the relationship to the middle cerebral artery more clearly than MRI. A second intrasellar meningioma is visible as an additional finding on both MRI and CTA (**arrows** in **A** and **B**).

## 4 Discussion

Ultra-high-resolution photon-counting CT can provide arterial, venous and osseous information in a single rapid acquisition and demonstrates the feasibility of incorporating PCCT into pre-operative neuronavigation workflows of resecting neurosurgical lesions, with the greatest added value in complex skull base meningiomas given the level of detail provided. A combined MRI-PCCT protocol should be developed and tested through an anatomical validation study followed by a prospective, ideally multicenter, phase II implementation study, which together should determine whether PCCT can be adopted as the modality of choice for all neurosurgical navigation scans.

An important consideration is that the clinical value of PCCT is likely greatest in the preoperative neuronavigation setting rather than during initial diagnosis or routine follow-up. While lesion detection and characterization can often be achieved adequately with contemporary MRI and conventional CT, surgical planning requires a more detailed understanding of the anatomical relationships between pathology and adjacent vascular and osseous structures. The chosen surgical approach is based on this information, including exposure, devascularization strategy, and the avoidance of vascular injury. Therefore, the ability of PCCT to provide UHR visualization of anatomical structures within a single acquisition and low-noise level may address a specific need for neuronavigation imaging, although whether the incremental anatomical detail translates into changes in surgical planning or intraoperative management was not directly assessed in this study.

Our findings build on rapidly expanding knowledge, characterizing the clinical potential of PCCT for neuronavigation. Unlike conventional energy-integrating detectors (EID), photon-counting detectors directly convert incident X-ray photons into electrical signals, eliminating electronic noise and enabling reconstruction at ultra-high spatial resolution with intrinsically spectral data (Willemink et al., 2018). Early *in-vivo* human studies established the feasibility and image quality of PCCT of the brain (Pourmorteza et al., 2017) and demonstrated superior depiction of the cervical and intracranial vasculature compared with conventional CT angiography [10.13]. Subsequent reviews have catalogued an expanding range of neuroradiological applications, from improved grey-white matter differentiation to high-fidelity vascular and osseous imaging (Douek et al., 2023; Benson et al., 2024). The substantially lower radiation exposure of the UHR CTA protocol is attributable to its optimization for vascular imaging rather than assessment of brain parenchyma, whereas the neuronavigation CT protocol is designed to provide high-resolution depiction of intracranial anatomy and brain parenchymal structures, requiring higher dose settings. To our knowledge, the present cohort is the first to translate these technical gains into a dedicated neurosurgical navigation and planning workflow, in which the combination of pure-lumen angiographic reconstructions and sub-millimeter (down to 0.2 mm while avoiding excessive noise/dose levels) osseous detail is of potential immediate operative relevance.

A central implication of our results is that a single ultra-high-resolution PCCT acquisition can supply the osseous, arterial, and venous information that is presently derived from several separate studies. Conventional neuronavigation typically relies on a post-contrast multi-slice CT for bony landmarks and registration, supplemented by MRI and, where vascular detail is required, MR angiography or venography or CTA (Watanabe et al., 1987; Orringer et al., 2012; Enchev, 2009; Kapsalaki et al., 2012; Choi et al., 2007). PCCT condenses much of this information into one rapid and more detailed examination: arterial and venous lumina are resolved with pure-lumen reconstructions while bone is simultaneously depicted at voxel sizes approaching 0.2 mm. The speed advantage is not trivial — CT acquisition is roughly an order of magnitude faster than a comparable MRI protocol, which reduces motion artefact, improves tolerability in claustrophobic, pediatric or clinically unstable patients, and shortens the planning pathway. The resolution gain is particularly valuable for calcification, hyperostosis and intraosseous tumor extension, which are poorly characterized on MRI and only coarsely resolved on conventional CT, yet are potentially relevant for craniotomy planning in lesions such as intraosseous meningiomas with considerable hyperostosis (Ungureanu et al., 2025; Agrawal et al., 2007).

It should be noted that MRI studies in this cohort were frequently obtained at referring hospitals and therefore varied in protocol and image quality. These external MRI datasets were not routinely repeated at our institution, which limits direct comparison between MRI and PCCT in some cases.

An important question is how PCCT should be integrated into the preoperative workflow. Based on our current experience, we do not envision PCCT replacing MRI as the primary modality for lesion characterization. MRI remains indispensable for the assessment of tumor extent, intra-axial infiltration, edema, cranial nerve involvement, diffusion characteristics, and advanced functional imaging. Rather, the greatest value of PCCT appears to be as complement to MRI as neuronavigation. In such a strategy, diagnostic MRI would provide lesion-specific information, while a dedicated pre-operative PCCT acquisition would supply high-resolution osseous and vascular anatomy. Future studies should evaluate whether a combined MRI-PCCT protocol improves surgical planning and workflow compared with current MRI- and conventional CT-based navigation strategies.

The optimal integration of PCCT into pre-operative workflows is also likely to depend on the underlying pathology. For benign extra-axial lesions, particularly skull base meningiomas, a dedicated pre-operative PCCT acquisition may provide much of the additional anatomical information required for surgical planning when a recent high-quality diagnostic MRI is already available. Similarly, for vascular pathologies such as arteriovenous malformations, aneurysms, and dural arteriovenous fistulae, the ability of PCCT to depict both vascular anatomy and surrounding osseous structures suggests a potentially more prominent role within the pre-operative workup. In contrast, for malignant intracranial tumors, including metastases and high-grade gliomas, MRI is likely to remain indispensable immediately prior to surgery, except when the vascular anatomy demands more detailed vascular imaging. Detection of interval tumor progression, multifocal disease, new lesions, edema, and infiltrative growth patterns may substantially influence treatment decisions and are not adequately assessed by CT-based imaging alone. Consequently, the role of PCCT as a standalone neuronavigation modality may be greatest for selected benign and vascular lesions, whereas a combined MRI-PCCT strategy will probably remain necessary for many malignant pathologies.

The advantages of PCCT were most evident in skull base meningiomas, where operative morbidity is closely tied to vascular anatomy (Ungureanu et al., 2025). PCCTA delineated dural arterial pedicles and tumor feeders that were not reliably seen on conventional CT or MR angiography, providing anatomical information that may be clinically relevant to surgical planning, including assessment of the surgical approach, devascularization strategy, and the potential feasibility and targeting of pre-operative embolization. However, the present study did not prospectively assess whether these findings altered surgical planning or management. High-resolution imaging also has the potential to reliably depict clinically important arterial variants, such as the meningo-ophthalmic artery, the recurrent meningeal artery and a persistent stapedial artery, whose recognition is essential to avoid ophthalmic and middle-ear complications during devascularization or endovascular treatment. On the venous side, PCCTV revealed a far more detailed venous tree, allowing identification of the dominant draining veins, the relationship of the lesion to the dural venous sinuses, and bridging veins at risk during retraction; preservation of these structures is a recognized determinant of post-operative venous infarction, particularly in posterior fossa and parasagittal approaches, which was not directly demonstrated in the present cohort. Detailed depiction of cavernous sinus involvement further permits correlation with established frameworks such as the Knosp grading system. To make these potential gains reproducible in routine practice, vascular reporting should be standardized: a structured checklist of the arterial feeders, anatomical variants and venous pathways relevant to each surgical approach would help radiologists and neurosurgeons convey the operatively critical findings to the surgical team (Beutler et al., 2024).

These observations also help to define which patients stand to benefit most. The incremental value of PCCT was greatest in anatomically complex, hypervascular or intraosseous lesions — principally skull base meningiomas, but also other vascularized tumors and vascular pathologies such as arteriovenous malformations, intracranial aneurysms even with perforating artery involvement, all of which were represented in our cohort. For simpler convexity lesions the additional vascular detail may be less decisive, although the workflow and acquisition-time advantages still apply. As with any iodinated-contrast, ionising-radiation examination, candidate selection should account for renal function, contrast allergy and cumulative dose, particularly in younger patients, even though photon-counting detectors offer favorable dose efficiency relative to conventional CT (Willemink et al., 2018; Douek et al., 2023).

Taken together, these findings support further prospective evaluation of ultra-high-resolution PCCT for neurosurgical navigation imaging. We therefore propose a staged pathway. First, an anatomical validation study should formally correlate PCCT-defined arterial feeders, variants and venous anatomy with the intraoperative findings taken as the reference standard, supplemented where available by digital subtraction angiography, to establish diagnostic accuracy. Building on this, a prospective phase II implementation study should evaluate PCCT-based navigation across consecutive neurosurgical candidates, with pre-specified endpoints including the rate of clinically relevant additional findings over conventional CT and MRI, the proportion of cases in which surgical planning is altered, navigation registration accuracy, acquisition and workflow time, radiation and contrast dose, and surgical safety outcomes. A multicenter design would be required to demonstrate generalizability beyond a single high-volume center and to provide the evidence base needed to establish PCCT as the modality of choice for all navigation scans in neurosurgery and to investigate its clinical utility.

This study has several limitations. Most importantly, the cohort is subject to substantial selection bias, as PCCT was performed in selected patients rather than consecutively, depending on clinical availability, and the cohort was enriched for large, anatomically complex, vascular and skull base lesions. It is a single-center, observational cohort reported in accordance with the STROBE guideline (von et al., 2008), and the sample, although spanning three years, is modest and heterogeneous, which limits subgroup inference. The present study was not designed to determine whether PCCT improves surgical safety, extent of resection, or functional outcomes and should be regarded primarily as hypothesis-generating rather than as evidence of clinical superiority or generalizability. As the operative plan for each patient was documented before any structured comparison of PCCT with conventional imaging took place, this study cannot establish that PCCT changed management. Demonstrating a causal relationship between PCCT-based planning and surgical outcomes will require prospective comparative studies. Because our standard clinical CT scanner is not a PCCT system, most patients receive their pre-operative CT on conventional EID-CT. Scanning clinical patients on the separate PCCT system requires workflow adjustments and so PCCT was performed only in selected patients throughout the years depending on clinical availability. This contributes to the modest sample size and heterogeneity of the cohort. The additional findings of PCCT compared to MRI have not yet been validated against an intraoperative or angiographic reference standard, so the clinical impact of the extra anatomical detail remains to be quantified. Furthermore, the MRI scans used for comparison were not uniformly state-of-the-art. As a tertiary referral center, many patients are referred with MRI studies acquired at peripheral hospitals using varying scanning protocols and scanner generations. In routine clinical practice, these MRIs are generally not repeated at our institution. Instead, only a neuronavigation scan is acquired preoperatively. This may have influenced comparisons between PCCT and MRI in some cases. Hard surgical outcomes, such as venous infarction or extent of resection, were not endpoints. Importantly, although PCCT demonstrated additional vascular and osseous anatomical detail with potential relevance to operative planning, we did not prospectively record whether these findings changed the planned surgical approach, sequence of devascularization, decision regarding pre-operative embolization, or intraoperative management on a case-by-case basis. Accordingly, these potential clinical benefits should be considered hypothesis-generating rather than demonstrated outcomes of this study. Additionally, unblinded side-by-side reviews may inflate apparent added value of PCCT, which is one of the reasons the series is presented as hypothesis-generating rather than definitive. Future prospective studies should incorporate case-level assessment of changes in surgical planning and intraoperative decision-making, with correlation of PCCT findings against operative and, where applicable, angiographic findings. Similarly, although PCCT acquisition was rapid, workflow advantages should not be inferred from the present study, as acquisition time, post-processing time, overall preparation time, and the need for additional imaging were not systematically measured or compared with the current workflow. Finally, PCCT scanners remain costly and unevenly available and realizing the workflow benefits in routine practice will require capital investment and a radiological learning curve. These limitations will be addressed by the proposed validation and phase II implementation studies are designed to address.

## 5 Conclusion

Ultra-high-resolution photon-counting CT can provide arterial, venous and osseous information in a single rapid acquisition and demonstrates the feasibility of incorporating PCCT into pre-operative neuronavigation, with potentially greatest added value in complex skull base meningiomas. These findings are preliminary and should be considered hypothesis-generating rather than evidence that PCCT can replace routine multislice CT or become the preferred modality for neurosurgical navigation. A combined MRI-PCCT protocol should be developed and tested through an anatomical validation study followed by a prospective, ideally multicenter, phase II implementation study, which should determine the diagnostic accuracy, clinical utility and potential role of PCCT in neurosurgical navigation.

## Ethics declaration

The authors have NOT obtained informed consent from participants or their legal representatives. The Medical Ethics Review Committee of Erasmus MC reviewed the study and determined that it did not fall within the scope of the Dutch Medical Research Involving Human Subjects Act (WMO). Consequently, formal ethical approval was not required (MEC-2026-0324).

This study was performed in compliance with relevant laws, regulatory frameworks and guidelines where the research took place. This study was conducted in accordance with non-WMO. The Medical Ethics Review Committee of Erasmus MC, Rotterdam, the Netherlands (METC Erasmus MC) granted an exemption. The authors provided the following explanation: The Medical Ethics Review Committee of Erasmus MC reviewed the study and determined that it did not fall within the scope of the Dutch Medical Research Involving Human Subjects Act (WMO). Consequently, formal ethical approval was not required (MEC-2026-0324).

## Declaration of competing interest

The authors declare that they have no known competing financial interests or personal relationships that could have appeared to influence the work reported in this paper.
