# Supplementary material for: Pre-operative visualization of cerebral vasculature using photon-counting CT ultra-high resolution pure lumen imaging
Source: Brain Spine. 2026 Sep 25;6:107451. doi: 10.1016/j.bas.2026.107451 (PMC13635442; doi:10.1016/j.bas.2026.107451)
Supplement: Supplementary file 1 [file mmc1.docx]

**Supplementary Appendix**

**Supplementary Appendix: Figure 1. Case #2.** Sphenoid, lateral orbital wall meningioma.

Post-contrast T1-weighted MRI (**A**) depicts the enhancing meningioma in the right sphenoid, lateral orbital wall region. UHR CTA with 0.2 mm slices (**B**) shows upward displacement of the right MCA (**arrow**). A matched-level MIP reconstruction (**C**) more clearly demonstrates the relationship between the MCA and the tumor, and additionally shows displacement of the deep perforating arteries, with left-right configuration differences indicated (**circles**). A 40 keV monoenergetic reconstruction of the CTA (**D**) provides optimal contrast and reveals a centrally located, dilated enhancing arterial structure within the tumor (**arrow**).

**Supplementary Appendix: Figure 2. Case #3.** Right sphenoid, lateral wall cavernous sinus dermoid cyst adjacent to the middle cerebral artery (MCA).

Axial and sagittal post-contrast MRI (**A–B**) show the intrinsically T1-hyperintense dermoid due to its fat content (**A**: *), with the MCA indicated (**B**: arrow). Axial 10mm MIP PCCT CTA and matched sagittal 0.2mm reconstructions (**C–D**) depict the low-attenuation dermoid in relation to the adjacent arterial structures, with inferior calcifications visible on the high-resolution sagittal image (**D**: circle). This case highlights the ability of PCCTA to distinguish tumor tissue from adjacent arteries more clearly than conventional imaging, thereby improving visualization of the vascular anatomy.

**Supplementary Appendix: Figure 3. Case #4.** Planum sphenoidale, tuberculum sellae meningioma.

T2-weighted MRI (**A**) shows displacement of both ACA (**arrows**) and conspicuous venous drainage vessels along the posterior and lateral margins of the mass (**circle**). UHR CTA with 0.2 mm slices (**B**) demonstrates a more detailed configuration, as does the standard neuronavigation CT. (**C**) Bone-subtracted CTA MIP reconstruction (**D**) depicts arterial supply entering from the inferior aspect of the tumor (**white arrow**) and venous drainage coursing superiorly with posterior outflow towards the cavernous sinus (**black arrows**).

**Supplementary Appendix: Figure 4. Case #5.** Spheno-orbital, lateral wall cavernous sinus meningioma.

Post-contrast T1w MRI (**A**) and post-contrast CT (**B**) acquired with the standard clinical neuronavigation protocol on PCCT depict the avidly enhancing tumor. UHR CTA (**C**) with 0.2 mm slices shows the origin of the ophthalmic artery, and UHR CTA 5.0 mm MIP reconstruction (**D**) demonstrates the inferolateral trunk (ILT) as a feeder to the meningioma on PCCTA (**arrow**).

**Supplementary Appendix: Figure 5. Case #6.** Giant ICA paraclinoid superior wall (ophthalmic artery) aneurysm.

Digital subtraction angiography (**A**) demonstrates the large, superior wall ICA aneurysm with a relatively small neck and a distinct posterior bleb (**circle**). Bone-subtracted UHR CTA (**B**) confirms the aneurysm morphology. 3D CTA reconstruction (**C**) further highlights the aneurysm. PCCTA enables neuronavigation at an image quality similar to DSA.

**Supplementary Appendix: Figure 6. Case #7.** Large anterior superior sagittal sinus meningioma with extensive hyperostosis.
Sagittal MIP T1w FSPGR post‑contrast (**A**) and CTA pure‑lumen imaging (**B**) show the meningioma with marked hyperostosis and intra‑osseous vascularity. The pure‑lumen CTA distinguishes the invaded versus open sinus segments (black arrow) and highlights pronounced osseous vascularization (white arrow). Axial T1w post‑contrast (**C**) and T2w MRI (**D**) depict sinus invasion in greater detail. CT bone reconstruction (**E**) outlines the extensive bony involvement. A second parietal meningioma is also present.

**Supplementary Appendix: Figure 7. Case #9.** Osteopetrosis with left-sided trigeminal neuralgia.

Ultra‑high‑resolution photon‑counting CT venography (PCCT CTV) of the skull base (**A**) demonstrates marked osseous sclerosis characteristic of osteopetrosis. High‑resolution sagittal T2 CUBE MRI (**B**) shows the course of the trigeminal nerve (arrow) from the pons to Meckel’s cave. Corresponding sagittal PCCT multiplanar reconstruction (**C**) in matching angulation illustrates the nerve trajectory within the densely sclerotic skull base. Pure‑lumen PCCT reconstruction (**D**) further enhances visualization of adjacent vascular structures. This case illustrates that MRI and PCCT provide complementary information for neuronavigation. MRI delineates cranial nerve anatomy and lesion extent, whereas PCCT provides high-resolution visualization of bone and vascular structures relevant to surgical planning.

**Supplementary Appendix: Figure 8. Case #10.** Recurrent pineocytoma.

Post-contrast 1.0 mm T1 MRI (**A**) depicts the enhancing recurrent tumor with associated hydrocephalus. A matched 0.4 mm CTV slice (**B**) emphasized the surrounding venous structures as well as the enhancing tumor in this scan phase. Sagittal 10 mm MIP CTV reconstruction (**C**) shows the internal cerebral veins (arrow) directly superior to the tumor, while coronal 10 mm MIP CTV reconstruction (**D**) demonstrates the basal veins (arrows) on both sides of the lesion in more detail.

**Supplementary Appendix: Figure 9. Case #11.** Metastasis (invasive mammary carcinoma), parieto-occipital region.

T1-weighted post-gadolinium MRI (**A**) and venous phase 40 keV monoenergetic PCCT reconstruction (**B**) demonstrate a large irregular enhancing mass in the left occipital lobe. High resolution 0.2 mm CTA reconstruction (**C**) reveals an arterial structure coursing along the inferior margin of the mass (**circle**), corresponding to a branch of the posterior cerebral artery. Matched 10mm MIP reconstruction (**D**) demonstrates more extensive arterial vasculature, as well as central microcalcifications within the mass (**arrow**).

**Supplementary Appendix: Figure 10. Case #12.** Right‑sided sphenoid-cavernous sinus meningioma.

Post-contrast MRI (**A**) shows a large mass centered in the right cavernous sinus with extension into the temporal compartment and through the superior orbital fissure toward the orbital apex, as well as invasion in the right sphenoid sinus. UHR bone-subtracted CTA (**B**) demonstrates the same extent of disease and additionally reveals luminal narrowing of the ICA (**arrow**). The CTV (**C**) shows clear enhancement of the mass and the coronal 40 keV CTV reconstruction (**D**) more distinctly depicts invasion of the sphenoid sinus and sparing of the sella turcica compared to MRI.

**Supplementary Appendix: Figure 11. Case #15.** Optic canal, cavernous sinus meningioma.

T1-weighted post-contrast MRI (**A**) shows a meningioma in the left superior orbital fissure (**arrow**). The 40 keV CTA PCCT reconstruction (**B**) and the bone window (**C**) depict the same lesion at the same level, with C demonstrating pronounced hyperostosis of the superior clinoid process and the greater sphenoid wing (**arrows**), while the foramen rotundum remains preserved.

**Supplementary Appendix: Figure 12. Case #17.** Sella turcica, cavernous sinus meningioma.

Coronal T2-weighted (**A**) and T1-weighted post-contrast (**B**) MRI show a large meningioma centered in the right cavernous sinus, with marked bulging of the sinus and extension into the sella and suprasellar cistern causing displacement of the optic chiasm (**arrow**), as well as invasion into the left cavernous sinus. The lumen of the right ICA is narrowed (**circle**). The standard CT for neuronavigation (**C**) as reference. The PCCT CTA MIP reconstruction (D) demonstrates vascular supply from the inferolateral trunk (**circle**) and encasement of the posterior communicating artery (**arrow**), which is invisible on MRI.

**Supplementary Appendix: Figure 13. Case #18.** Left insular astrocytoma.

Axial T2-weighted (**A**) and T1-weighted post-contrast MRI (**B**) show a large non-enhancing left insular mass traversed by branches of the MCA. A comparable configuration is seen on the UHR 0.2 mm PCCT (**C**). A coronal MIP reconstruction of the bone-subtracted CTA (**D**) demonstrates the MCA perforating arteries (**arrows**) only visible on PCCT, which are displaced medially by the mass.

**Supplementary Appendix: Figure 14. Case #19.** Lobulated left M1/lateral lenticulostriate aneurysms (unruptured).

Non-contrast TOF MRA (**A**) depicts the lobulated aneurysm at the right M1 segment (**circle**). The corresponding 0.2 mm CTA (**B**) shows the aneurysm at the same level (**circle**). A coronal bone-subtracted CTA MIP reconstruction (**C**) demonstrates an M2 branch arising medially from the aneurysm and coursing superiorly and laterally, with detailed MCA perforating arteries originating from this branch (**arrows**). A 3D reconstruction from the CTA (**D**) visualizes the aneurysm in a rotatable volumetric rendering.

**Supplementary Appendix: Figure 15. Case #20.** Right sphenoid wing intra-osseous meningioma.

Post-contrast T1-weighted MRI (**A**) shows marked hyperostosis of the right sphenoid wing with adjacent extra-osseous meningioma tissue extending temporally (**arrow**). The corresponding CTA images acquired on PCCT: 40 keV reconstruction (**B**), 0.2 mm bone reconstruction (**C**), and bone-subtracted reconstruction (**D**), depict the same lesions at the same level, demonstrating both the intra-osseous and extra-osseous components.
